# Knowledge and Awareness of the First Aid Management of Foreign Body Aspiration in Children Among the General Population: A Cross-Sectional Study in Saudi Arabia

**DOI:** 10.7759/cureus.68841

**Published:** 2024-09-06

**Authors:** Abdulaziz A AlRabiah, Tajah M Alaithan, Abdullah Alaithan, Amer S Alsaeri, Fatimah M Alenazi, Feras A Ageeli, Layla Al Khairat

**Affiliations:** 1 Emergency Medicine, King Saud University, Riyadh, SAU; 2 Medicine and Surgery, AlMaarefa University, Riyadh, SAU; 3 General Practice, Ministry of Health, Al-Hassa, SAU; 4 Medicine, Majmaah University, Majmaah, SAU; 5 Emergency Medicine, Diriyah Hospital, Riyadh, SAU; 6 Medicine, Jazan University, Jazan, SAU

**Keywords:** emergencies, first aid, foreign body aspiration, knowledge, pediatric

## Abstract

Objective: This study aims to assess the level of awareness and knowledge regarding the first aid management of foreign body aspiration (FBA) in children among various demographic groups.

Methods: A cross-sectional survey was conducted, involving 500 participants. The survey included questions about demographic information and knowledge of FBA management. The data were analyzed to identify significant differences in awareness levels based on gender, age, nationality, educational background, occupational status, and marital status.

Results: The study found significant differences in awareness levels across various demographic groups. Females (80.2%) demonstrated higher awareness compared to males (71.5%), with a significant p-value of 0.035. Participants aged 30-39 years had the highest awareness (83.6%), though the difference was not statistically significant (p=0.057). Nationality did not significantly affect awareness levels. However, educational background showed variations, with postgraduate degree holders having somewhat lower awareness (65.9%) compared to bachelor's degree holders (78.1%), though this difference was not significant (p=0.271). Occupational status had a significant impact, with military personnel displaying the highest awareness (100%) and business professionals the lowest (67.7%), with a significant p-value of 0.009. Marital status also influenced awareness, with married individuals showing higher awareness (82.8%) compared to single (74.4%) and divorced (65.5%) individuals, with a significant p-value of 0.040.

Conclusion: The study highlights the need for targeted educational programs to enhance first aid knowledge across different demographic groups. By addressing gaps in awareness through comprehensive training, especially among groups with lower awareness levels, we can improve the general population's ability to manage FBA incidents effectively, thereby reducing associated morbidity and mortality in children.

## Introduction

In recent years, there has been a rise in the occurrence of serious complications caused by the ingestion of hazardous objects, leading to airway foreign bodies becoming a common and urgent medical emergency in children. Commonly encountered items such as small toys, marbles, batteries, and erasers are often involved, with a notable frequency of ingestion observed for button batteries and magnets [1-4]. Young children, especially between one and three years old, are more susceptible to foreign body aspiration (FBA) due to their underdeveloped posterior molars and narrower airways that are still growing [5,6]. To better detect foreign body ingestion and FBA, healthcare professionals should be highly suspicious, recognize the diverse presentations, and consider it in children with non-specific symptoms like fever, cough, drooling, difficulty swallowing, and abdominal pain [3]. Children with signs of airway obstruction or a history suggesting dangerous foreign body ingestion should be treated as an emergency because it can be life-threatening and requires immediate intervention [3,7-9]. The symptoms and signs of FBA vary depending on the size, location, nature, and time since the foreign body entered the trachea or bronchi. Foreign bodies can lodge in various parts of the head and neck, including the nose, ears, throat, hypopharynx, bronchus, and esophagus [10,11]. Obstruction caused by foreign bodies can severely impair oxygenation and ventilation, potentially leading to hypoxic-ischemic brain injury, which is the most common cause of death in these situations [12]. To prevent FBA, it is crucial to educate parents on the importance of supervision and provide them with guidelines on preventing and managing FBA incidents. Public awareness should also be increased to facilitate timely diagnosis and appropriate management [13,14]. The implementation of stringent safety standards for toys and household items has been identified as essential in preventing FBA incidents, prompting calls for regulatory bodies to enforce stricter safety measures [15]. Advances in endoscopic techniques and retrieval tools have significantly enhanced the success rates and safety of foreign body removal procedures [16]. Additionally, the incorporation of simulation-based training for healthcare providers has proven beneficial in improving the skills necessary for the emergency management of FBA [17]. Recent studies also highlight the importance of a multidisciplinary approach involving pediatricians, otolaryngologists, and emergency medicine specialists to ensure comprehensive care and optimal outcomes for affected children [18]. Furthermore, community-based programs aimed at educating parents and caregivers about the risks and prevention strategies for FBA have shown to be effective in reducing the incidence of these emergencies [19]. Our primary objective is to comprehensively evaluate the level of knowledge and awareness regarding the first aid management of FBA within the general population. This assessment will delve into the understanding of recognition of signs and symptoms, appropriate first aid measures, and the importance of calling emergency services. Additionally, it will investigate knowledge of prevention strategies. This comprehensive assessment will provide valuable insights into the public's preparedness to handle FBA emergencies and inform targeted educational initiatives and public awareness campaigns to improve knowledge, skills, and confidence in managing these potentially life-threatening situations.

## Materials and methods

This cross-sectional study aimed to evaluate the level of knowledge and awareness regarding the first aid management of FBA among a sample of 500 participants from the general population in Riyadh, Saudi Arabia. A questionnaire (Appendices) was used as the research instrument, which was openly distributed through various social media platforms by all authors. Participants were encouraged to share the questionnaire link with their personal and professional contacts. Eligibility criteria included being at least 18 years old, residing in Riyadh, and expressing willingness to participate, without any gender restrictions. Prior to data collection, ethical approval was obtained from the Institutional Review Board of King Saud University in Riyadh (approval number: E-24-8944), and informed consent was obtained from all participants after providing a clear explanation of the study's objectives and eligibility criteria. The questionnaire consisted of three sections: the first section collected sociodemographic information, while the second section assessed knowledge and awareness of the first aid management of FBA. The questionnaire was available in both Arabic and English, and a meticulous validation process was conducted based on the methodology described by Bin Laswad et al. [20]. To ensure accurate translation, two native Arabic speakers independently translated the questionnaire, and a thorough review was performed to address any inconsistencies or potential misinterpretations. Data analysis was conducted using IBM SPSS Statistics for Windows, Version 25.0 (Released 2017; IBM Corp., Armonk, New York, United States), involving descriptive statistics to summarize responses and inferential statistics to explore relationships between variables. The results were presented through tables and text, with a p-value of less than 0.05 considered statistically significant.

## Results

Table 1 presents the demographic data of the participants. The gender distribution shows that out of the 500 participants, 144 were male (28.7%) and 358 were female (71.3%). In terms of age, the majority of participants fell into the 18-29 age group, with 268 participants (53.4%). The 30-39 age group had 128 participants (25.5%), followed by the 40-49 age group with 73 participants (14.5%). The 50-59 age group had 29 participants (5.8%), and there were four participants above the age of 60 (0.8%). In terms of nationality, the majority of participants were Saudi, with 461 participants (91.8%), while 41 participants were non-Saudi (8.2%). Regarding education, four participants had an elementary school education (0.8%), 13 participants had an intermediate school education (2.6%), 107 participants had a high school education (21.3%), 44 participants had a bachelor's degree (8.8%), and there were no participants with a postgraduate school education. In terms of occupational status, 138 participants were students (27.5%), 66 participants were teachers (13.1%), eight participants were in the military (1.6%), 24 participants were engineers (4.8%), 16 participants were professors (3.2%), 13 participants were lawyers (2.6%), 133 participants were in business (26.5%), 77 participants were in the medical field (15.3%), and 27 participants were unemployed (5.4%). Lastly, in terms of marital status, 258 participants were single (51.4%), 204 participants were married (40.6%), and the remaining participants did not provide their marital status.

**Table 1:** Demographic data of the participants. The data in the table has been presented as follows: N indicates frequency, and % indicates percentages.

Parameter		N	%
Gender	Male	144	28.7
	Female	358	71.3
Age (in years)	18-29	268	53.4
	30-39	128	25.5
	40-49	73	14.5
	50-59	29	5.8
	Above 60	4	0.8
Nationality	Saudi	461	91.8
	Non-Saudi	41	8.2
Education	Elementary school	4	0.8
	Intermediate school	13	2.6
	High school	107	21.3
	Bachelor degree	334	66.5
	Postgraduate school	44	8.8
Occupational status	Student	138	27.5
	Teacher	66	13.1
	Military	8	1.6
	Engineer	24	4.8
	Professor	16	3.2
	Lawyer	13	2.6
	Businessman/businesswomen	133	26.5
	Medical field	77	15.3
	Unemployed	27	5.4
Marital status	Single	258	51.4
	Married	204	40.6
	Divorced	29	5.8
	Widowed	11	2.2
Household income	Less than 10,000	268	53.4
	More than 10,000	234	46.6

The results from Table 2 highlight key questions regarding FBA and choking in children. Children aged 1-5 years are at the highest risk of aspirating foreign bodies, 312 (62.2%), followed by those less than one year old, 169 (33.7%). Both organic items like nuts and small plastic toys pose significant risks, 408 (81.3%). Most participants agree that children should not be offered peanuts before the age of four, 395 (78.7%), and talking while chewing increases aspiration risk, 444 (88.4%). The absence of choking is not always an assuring sign that a foreign body has been expelled, 353 (70.3%), and delaying removal if there are no symptoms is generally not advised, 350 (69.7%). While X-rays can detect many foreign bodies, 331 (65.9%), not all are visible. Most respondents would attempt to remove a foreign body from a child's mouth with their fingers, 343 (68.3%). For choking treatment, back slaps or abdominal thrusts are recommended if the child is choking and able to talk, 360 (71.7%), and even more so if the child cannot talk, 450 (89.6%). Medical attention should be sought even if symptoms subside shortly after an incident, 370 (73.7%).

**Table 2:** Related questions about foreign body aspiration and choking in children. The data in the table has been presented as follows: N indicates frequency, and % indicates percentage.

Parameter			N (%)
Age and risk factors	At which age are children at the highest risk of aspirating foreign bodies?	Less than one year	169 (33.7)
		1-5 years	312 (62.2)
		6-10 years	14 (2.8)
		More than 10 years	7 (1.4)
	Which items pose a risk of aspiration for children?	Organic like nuts	39 (7.8)
		Small plastic toys	46 (9.2)
		Both	408 (81.3)
		None	9 (1.8)
Prevention and dietary guidelines	Should children be offered peanuts before they turn four years old?	Agree	395 (78.7)
		Disagree	107 (21.3)
	Talking while chewing increases the risk of aspiration?	Agree	444 (88.4)
		Disagree	58 (11.6)
Symptoms and removal	Is the absence of choking an assuring sign that the foreign body has been expelled?	Agree	353 (70.3)
		Disagree	149 (29.7)
	If the foreign body causes no symptoms, is it okay to delay removal?	Agree	152 (30.3)
		Disagree	350 (69.7)
	Can X-rays detect all types of foreign bodies?	Agree	331 (65.9)
		Disagree	171 (34.1)
	Would you attempt to remove the foreign body inside the child's mouth with your fingers?	Agree	343 (68.3)
		Disagree	159 (31.7)
Choking treatment	Would you attempt to do back slaps or abdominal thrusts if the child is choking and able to talk?	Agree	360 (71.7)
		Disagree	142 (28.3)
	Would you attempt to do back slaps or abdominal thrusts if the child is choking and not able to talk?	Agree	450 (89.6)
		Disagree	52 (10.4)
Medical attention	Should you go to a hospital if there are no symptoms or if the symptoms subside shortly after?	Agree	370 (73.7)
		Disagree	132 (26.3)

Table 3 presents the level of awareness in relation to demographic data, highlighting that gender, age, and occupational status significantly influence awareness levels. Females, 287 (80.2%), demonstrated a higher level of good knowledge compared to males, 103 (71.5%), with a significant p-value of 0.035. Age-wise, participants aged 30-39 years, 107 (83.6%), had a higher level of good knowledge compared to other age groups, although the p-value was 0.057. Occupational status showed a significant variation in knowledge levels with students, 109 (79%), teachers, 54 (81.8%), and engineers, 20 (83.3%), having higher good knowledge percentages and a significant p-value of 0.009. Marital status also impacted knowledge levels, with single participants, 192 (74.4%), demonstrating higher good knowledge and a significant p-value of 0.040.

**Table 3:** Level of awareness in relation to demographic data. Correlation between knowledge and demographic data of the participants shows significant p-values of gender (p=0.035), occupational status (p=0.009), and marital status (p=0.040).

		Knowledge			
Parameter		Good (%)	Poor (%)	Total (%)	P-value
Gender	Male	103 (71.5)	41 (28.5)	144 (28.7)	0.035
	Female	287 (80.2)	71 (19.8)	358 (71.3)	
Age (in years)	18-29	195 (72.8)	73 (27.2)	268 (53.4)	0.057
	30-39	107 (83.6)	21 (16.4)	128 (25.5)	
	40-49	59 (80.8)	14 (19.2)	73 (14.5)	
	50-59	26 (89.7)	3 (10.3)	29 (5.8)	
	Above 60	3 (75)	1 (25)	4 (0.8)	
Nationality	Saudi	358 (77.7)	103 (22.3)	461 (91.8)	0.954
	Non-Saudi	32 (78)	9 (22)	41 (8.2)	
Education	Intermediate school	10 (76.9)	3 (23.1)	13 (2.6)	0.271
	High school	90 (81.1)	21 (18.9)	111 (22.1)	
	Bachelor degree	261 (78.1)	73 (21.9)	334 (66.5)	
	Postgraduate school	29 (65.9)	15 (34.1)	44 (8.8)	
	Student	109 (79)	29 (21)	138 (27.5)	
Occupational status	Teacher	54 (81.8)	12 (18.2)	66 (13.1)	0.009
	Military	8 (100)	0 (0)	8 (1.6)	
	Engineer	20 (83.3)	4 (16.7)	24 (4.8)	
	Professor	11 (68.8)	5 (31.5)	16 (3.2)	
	Lawyer	8 (61.5)	5 (38.5)	13 (2.6)	
	Businessman/businesswomen	90 (67.7)	43 (32.3)	133 (26.5)	
	Medical field	69 (89.6)	8 (10.4)	77 (15.3)	
	Unemployed	21 (77.8)	6 (22.2)	27 (5.4)	
	Single	192 (74.4)	66 (25.6)	258 (51.4)	
Marital status	Married	169 (82.8)	35 (17.2)	204 (40.6)	0.040
	Divorced	19 (65.5)	10 (34.5)	29 (5.8)	
	Widowed	10 (90.9)	1 (9.1)	11 (2.2)	
Household income	More than 10,000	181 (77.4)	53 (22.6)	234 (46.4)	0.865
	Less than 10,000	209 (78)	59 (22)	268 (53.4)	

## Discussion

The findings of this study highlight several key points regarding the knowledge and awareness of the first aid management of FBA among the general population and relate well to the emphasis on clinical vigilance discussed in the literature. McKinney et al. [3] address the prevalence, risks, and management of foreign body ingestion and FBA in children, stressing the importance of healthcare professionals maintaining high awareness of varying presentations and including it as a differential diagnosis in children presenting with non-specific symptoms. This aligns with our study's emphasis on the need for general awareness, particularly focusing on demographic factors that influence first aid knowledge. For instance, females demonstrated higher awareness levels, 287 (80.2%), compared to males, 103 (71.5%), and age also played a critical role, with individuals aged 30-39 years showing the highest awareness, 107 (83.6%) (p=0.035). Educational background and occupational status significantly impacted awareness, with military personnel demonstrating the highest awareness, 8 (100%), and business professionals the lowest, 90 (67.7%) (p=0.009). Additionally, marital status influenced awareness, with married individuals showing significantly higher awareness, 169 (82.8%), compared to single, 192 (74.4%), and divorced, 19 (65.5%), individuals (p=0.040).

Gregori et al. [4] further underscore the seriousness of foreign body injuries, particularly in otorhinolaryngological practice, noting that foreign body injuries in the nose can lead to long-term complications and highlighting the need for public education. This is consistent with our findings that stress the importance of demographic factors in awareness levels and the need for comprehensive education.

Kiliçaslan et al. [5] emphasize the critical role of parental education in preventing FBA, noting that increased educational levels among Turkish mothers correlated with higher awareness of FBA's lethality and the importance of preventive measures and first response. This finding is in line with our study, where a higher educational background significantly impacted FBA awareness.

Higuchi et al. [14] highlight significant gaps in parental knowledge regarding FBA, identifying risk factors such as being a mother with a child younger than 12 months old or being a first-time mother. This complements our findings that various demographic factors, including age and marital status, play crucial roles in awareness levels.

Alshehri et al. [12] found that students in Jeddah, Saudi Arabia, had varied levels of knowledge about FBA, with significant differences based on gender and experience. The study reported high awareness that small toys could cause choking (93.2% among males and 94% among females), but lower recognition that sudden cough is a sign of choking (40.8% among males and 33% among females). The knowledge that batteries are dangerous if swallowed was significantly higher among females (74.2%) than males (56.2%) (p<0.001). This aligns with our findings on the impact of gender on FBA awareness, showing similar trends in differing demographic contexts.

Salman et al. [13] identify gaps in parental knowledge regarding FBA symptoms and first aid management, noting that 49.7% of parents recognized breathlessness as a symptom, while fewer recognized vomiting (7.3%), hand clutched to throat (6.6%), color change (9.9%), and voice change (10.6%). As a first aid measure, 66.2% knew about back slaps, but only 2% were aware of abdominal thrusts. Literate parents were significantly more aware of the risks associated with certain foods and behaviors compared to illiterate parents (p<0.05). These findings are consistent with our study's results, where education level significantly impacted awareness of FBA.

These demographic insights from McKinney et al. [3], Gregori et al. [4], Kiliçaslan et al. [5], Higuchi et al. [14], Alshehri et al. [12], and Salman et al. [13] underscore the vital role of healthcare professionals in providing education to parents and the need for broader public education campaigns. Combining these perspectives reinforces the idea that both professional healthcare education and public awareness are crucial in preventing and managing FBA incidents in children, leading to improved patient outcomes. 

One of the limitations of this study is its reliance on self-reported data from participants, which may be subject to recall bias or inaccuracies in the self-assessment of knowledge and awareness. Additionally, the use of social media for survey distribution may have introduced selection bias, as it could have skewed the sample towards individuals who are more active online and possibly more educated or informed than the general population. Finally, the study was conducted in a specific geographical region, which may limit the generalizability of the findings to other populations or regions with different demographic characteristics.

## Conclusions

This study underscores the critical importance of enhancing public awareness and education regarding the first aid management of FBA in children. The analysis reveals significant disparities in awareness levels across different demographic groups, notably gender, age, and occupational status. Females and individuals aged 30-39 years demonstrated higher levels of knowledge, while occupational status notably influenced awareness, with military personnel showing the highest awareness levels. The findings suggest that tailored educational programs targeting specific demographic groups, particularly those with lower awareness levels, could substantially improve the general population's preparedness to handle FBA incidents effectively. By addressing these gaps through comprehensive and accessible first aid training, we can mitigate the risks associated with FBA, ultimately reducing the incidence of preventable morbidity and mortality among children.

## Appendices

**Table 4:** Study questionnaire.

Questionnaire
1. Age (in years)
18-29
30-39
40-49
50-59
More than 60
2. Gender
Male
Female
3. Marital status
Single
Married
Divorced
Other
4. Nationality
Saudi
Non-Saudi
5. Level of education
Illiterate
Elementary
Intermediate
Secondary
College
Postgraduate
6. Occupation
Student
Teacher
Military
Engineer
Professor
Lawyer
Businessperson
Medical field
Unemployed
7. Monthly income
<10,000 SR
≥10,000 SR
8. Highest risk age for foreign body aspiration
<1 year
1-5 years
6-10 years
>10 years
9. Shouldn't offer peanuts until four years old
Agree
Disagree
10. Absence of choking is assuring
Agree
Disagree
11. Delay removal if no symptoms
Agree
Disagree
12. Talking while chewing can lead to aspiration
Agree
Disagree
13. X-rays detect all foreign bodies
Agree
Disagree
14. Items children are at risk of aspirating
Organic (like nuts)
Non-organic (like toys)
Both
None
15. Remove the foreign body from the child's mouth with fingers
Yes
No
16. Do back slaps/abdominal thrusts if the child is choking but able to talk
Yes
No
17. Do back slaps/abdominal thrusts if the child is choking and unable to talk
Yes
No
18. Go to a hospital if there are no symptoms or symptoms subside
Yes
No
